# Associations between PFAS occurrence and multimorbidity as observed in an electronic health record cohort

**DOI:** 10.1097/EE9.0000000000000217

**Published:** 2022-07-14

**Authors:** Cavin K Ward-Caviness, Joshua Moyer, Anne Weaver, Robert Devlin, David Diaz-Sanchez

**Affiliations:** Center for Public Health and Environmental Assessment, US Environmental Protection Agency, Chapel Hill, NC.

**Keywords:** PFAS, Multimorbidity, Electronic health records, PFOA, PFHpA, Chronic disease

## Abstract

Per and polyfluoroalkyl substances (PFAS) are associated with health outcomes ranging from cancer to high cholesterol. However, there has been little examination of how PFAS exposure might impact the development of multiple chronic diseases, known as multimorbidity. Here, we associated the presence of one or more PFAS in water systems serving the zip code of residence with chronic disease and multimorbidity.

**Methods:**

We used data from the unregulated contaminant monitoring rule 3 to estimate exposure to PFAS for a random sample of 10,168 patients from the University of North Carolina Healthcare System. The presence of 16 chronic diseases was determined via. their electronic health records. We used a logistic regression model in a cross-sectional study design to associate the presence of one or more PFAS with multimorbidity. Models were adjusted for age, race, sex, smoking status, socioeconomic status, and 20 county-level confounders.

**Results:**

There were four PFAS found in water systems that served at least one zip code represented in our patient data: PFOA, PFHpA, PFOS, and PFHxS. Exposure to any PFAS was associated with a odds ratio of 1.25 for multimorbidity (95% confidence interval = 1.09, 1.45). Among the chronic diseases with at least 300 cases, we observed associations with dyslipidemia, hypertension, ischemic heart disease, and osteoporosis.

**Conclusion:**

Exposure to PFAS is associated with a range of chronic diseases as well as multimorbidity. Accounting for the joint impacts of PFAS on multiple chronic conditions may give an increasingly clear picture of the public health impacts of PFAS.

**What this study adds**

This study estimates the association between multiple PFAS chemical species and both multimorbidity as well as individual chronic diseases. To the author’s knowledge this is the first manuscript to use multimorbidity as an outcome for PFAS health effects. This manuscript is also among the first to use electronic health records to study PFAS, and thus presents a new resource to apply to the study of their health effects. Electronic health records present an efficient means of obtaining large, well phenotyped study cohorts with characteristics, for example, rare diseases that can be difficult to study using general population-based cohorts.

## Introduction

Per and polyfluoroalkyl substances (PFAS) are synthetic compounds whose chemical structure resembles hydrocarbons (with various functional groups) with multiple (poly) or all (per) hydrogens replaced with fluorine. Replacing the hydrogens with fluorine gives the PFAS family of chemicals several properties including heat resistance, hydrophobicity, and a long half-life that have made them extremely useful for a variety of industrial and household applications. It is estimated that PFAS are used in more than 200 industrial applications.^1^ It is estimated that over 98% of the US population has one or more detectable PFAS chemicals in their blood stream.^2^ As many PFAS have half-lives of multiple years,^3^ this high prevalence of exposure means that the public health implications of exposure to PFAS will persist for many years.

During the heights of their production and usage, many facilities discharged PFAS chemicals directly into waterways or into the atmosphere. Drinking water remains a prevalent route of exposure for PFAS chemicals, although exposure via. airborne dust, food packaging, and cookware may all remain relevant to this day.^4^ PFAS exposure is associated with multiple health outcomes including cancer,^5^ lipid metabolism,^6,7^ thyroid disruption,^8,9^ cardiovascular disease,^10,11^ and kidney disease.^12,13^ In addition, mechanistic studies have probed the molecular actions that lead to such a wide array of health outcomes for PFAS and have shown that PFAS are associated with increases in markers of oxidative stress.^14^ PFAS may also exert many of their health effects via. disruption of the nuclear receptor family, in particular peroxisome proliferator-activated receptor alpha (PPARα).^15,16^ However, in considering the mechanism of action of PFAS, it is important to note that this diverse chemical class has an array of molecular effects, which we are continuing to profile and understand.^16,17^

One resource that has not been widely applied to PFAS research is electronic health records (EHRs). The use of EHRs in research has accelerated over the past decade, and they are now widely used within environmental epidemiology, particularly for the study of air quality^18,19^ and social determinants of health.^20^ One of the main advantages of EHRs is their broad assessment of health outcomes. This depth of clinical phenotyping enables comprehensive studies of health outcomes using a single resource. In the case of PFAS exposures, EHRs can facilitate a shift from examining exposure effects on individual chronic outcomes to examining the impact of PFAS on multimorbidity, that is, the occurrence of multiple chronic diseases.^21^ As the prevalence of multimorbidity may be increasing in the US population,^22^ this is an important health outcome to understand and distinguish from examining individual chronic diseases. Given the broad swath of health effects associated with PFAS exposures, examining its associations with multimorbidity may give a unique insight into the public health concerns of this long-lived chemical class. Here, we will use EHRs contained within the EPA Clinical and Archived records Research for Environmental Studies (CARES) resource^23^ to examine associations between PFAS exposure and multimorbidity in North Carolina.

## Methods

### Study population

Data for this project came from the EPA CARES resource, a collection of electronic health records from the University of North Carolina Healthcare System that have been integrated with environmental exposure data for use in environmental health studies.^19,23^ Patient addresses were considered successfully geocoded at the street or zip code level. For this study, we used a random sample of North Carolina residents who had an interaction with a University of North Carolina Healthcare System affiliated hospital or clinic at least once between January 1, 2004, and December 31, 2016, that generated an electronic health record. To limit exposure misclassification by individuals changing addresses, we restricted to those only reporting a single address for their observed time. This study was approved under IRB 20-0123, and informed consent was waived. Chronic disease was determined using ICD-9 and ICD-10 codes for 16 chronic diseases based on a published definition of multimorbidity suitable for EHRs.^21^ We also included liver disease due to previous associations between PFAS exposure and liver dysfunction.^24,25^ All ICD-9 and ICD-10 codes came from the electronic health record and were filtered for billing codes before determination of chronic diseases or multimorbidity. ICD-9 and ICD-10 code definitions for the 16 chronic diseases can be found in eTable 1 (http://links.lww.com/EE/A192).

### PFAS exposure

The primary exposure metric used in this study is whether or not a water system serving the zip code of residence tested positive for any PFAS chemical species or not. PFAS presence in water systems was determined using publicly available data on water testing done under the Third Unregulated Contaminant Monitoring Rule (UCMR3; https://www.epa.gov/dwucmr/third-unregulated-contaminant-monitoring-rule). The UCMR3 was performed under authority granted by the 1996 Safe Drinking Water Act which requires regular (every 5 years) monitoring of up to 30 unregulated contaminants in public water systems. The UCMR3 set forth to monitor 28 chemicals and 2 viruses, including 6 PFAS. For each PFAS, a minimum reporting level (MRL) was set based on the parameters of the measurement assay for that PFAS. Monitoring for PFAS was performed in all public water systems serving more than 10,000 individuals, as well as a representative sample of public water systems serving 10,000 or fewer individuals. All PFAS were assessed using an identical, published solid phase extraction and liquid chromatography/tandem mass spectrometry method.^26^ Testing for the UCMR3 was done between 2013 and 2015. Concentrations for each PFAS at or above the MRL were reported, with concentrations below the MRL simply indicated as being less than the MRL. As the UCMR3 only provided the zip codes served by each public water system tested, we linked potential exposure with patients based on their geocoded zip code. Geocoding procedures for patients were identical to those used in previous analyses of EPA CARES.^23^ There were four PFAS which were observed above the MRL for at least one zip code in which a study participant also resided: perfluorooctanoic acid (PFOA; 38 zip codes; MRL = 0.02 µg/L), perfluoroheptanoic acid (PFHpA; 60 zip codes; MRL = 0.01 µg/L), perfluorooctanesulfonic (PFOS; 10 zip codes; MRL = 0.04 µg/L), and perfluorohexanesulfonic (PFHxS; 10 zip codes; MRL = 0.03 µg/L). Given the lack of a continuous distribution of observed PFAS concentrations (eFigure 1; http://links.lww.com/EE/A192), we classified each zip code as having the presence of a tested PFAS or not based on a recorded test above the minimum reporting level at least once. This binary classification approach mirrors that taken by previous analyses of PFAS using electronic health records.^27^ Although testing for the UCMR3 (2013–2015) occurred toward the end of our study period, the long half-lives of PFAS chemicals suggests that historical concentrations were if anything higher in the past.

### Statistical approach

We defined multimorbidity as the occurrence of two or more of the chronic conditions listed in eTable 1 (http://links.lww.com/EE/A192). We examined associations between PFAS exposure and multimorbidity using logistic regression while adjusting for age, sex, race, smoking status (current/former/never), percent urbanicity at the census block group, median household value at the census block group, percent homes under the federal poverty line at the census block group, and percent of homes receiving public assistance at the census block group. All census variables were taken from the 2010 census. We also adjusted for 19 county-level indicators of socioeconomic status and access to health care from the County Health Rankings.^28^ The county-level variables and their definitions can be found in eTable 2 (http://links.lww.com/EE/A192). We examined associations between multimorbidity and the presence of a PFAS chemical in at least one water system serving the zip code of residence. We also examined associations with PFOA and PFHpA individually in separate models. We did not examine PFOS and PFHxS separately due to the limited number of zip codes (10) served by a water system that tested positive for either of them. To examine potential compounding effects of multiple PFAS, we also examined associations between none versus at least one of PFOA or PFHpA as well as between none versus both PFOA and PFHpA. We again did not include PFOS and PFHxS in these examinations as only a few public water systems serving a limited set of zip codes tested positive for these PFAS.

We stratified the study cohort on race, sex, and median household income at the census block group level to examine potential differences by self-identified race and sex as well as neighborhood socioeconomic status. In addition, we used a cumulative link model (logit link) to examine the association between PFAS exposure and an additional chronic condition as opposed to strictly just the presence or absence of multimorbidity. Cumulative link models model the probability of a transition from one category to another, for example, being diagnosed with an additional chronic disease, without the strong assumption that the difference between categories is the same. This makes them a reasonable choice for modeling multimorbidity where, for example, the difference between having 2 chronic diseases versus 3 is not necessarily the same as the difference between 3 versus 4. The confounder adjustment for the cumulative link model remained the same as the confounder adjustment for the logistic regression model. To limit the impact of outliers on associations from the cumulative link model we grouped 5 or more comorbidities into a single category.

As a secondary analysis, we also examined associations between PFAS exposure and individual chronic diseases. To have sufficient sample size, we selected only those chronic diseases with greater than 300 cases in our study cohort. We also examined an additional adjustment for the percent public water usage at the county level using data obtained from US Geological Survey^29^ to see if this modified observed associations. We also examined sensitivity to our definition of a positive test by requiring that a public water system test positive for a PFAS at least twice before that PFAS was declared present. Owing to the high prevalence of missing smoking data, we used multiple imputation as implemented in the *mice* R package^30^ to impute smoking status to see if this altered the associations as done for previous analyses for EPA CARES.^19^ Covariates used in the imputation analysis were the same as those used for the association models described above. All analyses were performed using R version 4.0.3^31^ and are reported as the odds ratio (OR) and associated 95% confidence interval (95% CI). The R package *ordinal* was used to perform the cumulative link models.^32^

## Results

**Figure 1.**
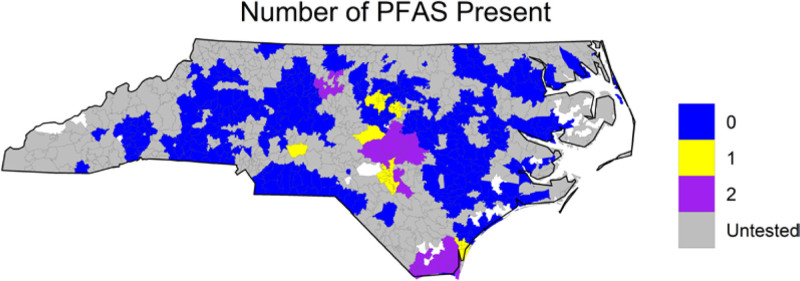
Number of PFAS present in NC. Shown in color is the number of PFAS present in North Carolina. A PFAS was determined as present if a public water system serving that zip code tested positive (above minimum reporting limit) for that PFAS. No zip code tested positive for more than 2 PFAS.

The spatial distribution of zip codes served by a public water system testing above the MRL for at least one PFAS is given in **Figure 1**. The spatial distributions of PFOA and PFHpA testing are given in eFigure 2 (http://links.lww.com/EE/A192). There were 10,168 individuals in our final study cohort. Of these individuals 4228 resided in a zip code served by a public water system that tested positive for at least 1 PFAS. There were 2966 study participants residing in a zip code that tested positive for PFOA and 2,096 for PFHpA. Our study cohort had a mean age of 55.0, was 61.2% female, and 22.9% self-identified as Black or African American (**Table 1**). There were 6442 participants who were not diagnosed with any of the 16 chronic diseases examined and 2242 who had two or more chronic diseases—our definition of multimorbidity as described in the Methods. The frequencies of the chronic diseases can be found in eTable 3 (http://links.lww.com/EE/A192). The characteristics of our study cohort, which was restricted to those with just one reported address, matched those of the entire population (eTable 4; http://links.lww.com/EE/A192), indicating that the restriction to one address did not bias the analyses, although it likely decreased exposure misclassification.

**Table 1.** Study cohort description

	Mean	SD
Age (yrs)	55.0	18.2
Percent urbanicity	69.2	39.2
Median home value ($)	210,768	119,610
Percent households below federal poverty level	16.1	14.2
Percent of homes receiving public assistance	1.77	2.77
Observation time (y)	7.29	4.37
	**N**	**%**
Females	6,221	61.2
Males	3,947	38.8
Race—White	6,697	65.9
Race—Black	2,333	22.9
Race—Other	1,138	11.2
Never smoker	2,519	24.8
Former smoker	1,271	12.5
Current smoker	768	7.55
Unknown smoking status	5,610	55.2
Multimorbidity	2,242	22.0
PFOA present	2,966	29.2
PFHpA present	2,096	20.6
Any PFAS present	4,228	41.6

Statistical description of the study cohort (N = 10,168). Percent urbanicity, median home value, percent households below federal poverty level, and percent homes receiving public assistance all assessed using the 2010 US Census at the block group level.

SD indicates standard deviation.

### PFAS and multimorbidity

We observed significant associations between the presence of any PFAS in a water system serving a zip code and multimorbidity. The presence of any PFAS was associated with a 25% higher prevalence of multimorbidity (OR = 1.25, 95% CI = 1.09, 1.45; **Table 2**). This association was robust to adjustment for public drinking water usage (1.22, 95% CI = 1.06, 1.41) as well as increasing the stringency of the definition of positive presence of PFAS by requiring two tests above the MRL (OR = 1.27, 95% CI = 1.10, 1.47). We also observed associations when using a cumulative link model where the presence of any PFAS was associated with a 24% increase in the prevalence of one additional chronic condition (OR = 1.24, 95% CI = 1.10, 1.39; **Table 3**).

**Table 2.** Associations between PFAS and multimorbidity via. logistic regression

Exposure	Main model	Min 2 Positive Tests	County Water Use Adjusted
Any PFAS	1.25 (1.09, 1.45)	1.27 (1.10, 1.47)	1.22 (1.06, 1.41)
PFOA	1.30 (1.12, 1.52)	1.24 (0.98, 1.56)	1.30 (1.12, 1.51)
PFHpA	1.20 (0.97, 1.47)	1.27 (1.01, 1.60)	1.13 (0.92, 1.40)

Results of the association between PFAS exposure and multimorbidity using logistic regression are presented as the odds ratio with the 95% confidence interval in parentheses. In the “Exposure” column Any PFAS represents exposure to any of the four PFAS found in NC while PFOA and PFHpA refer to exposure to those specific PFAS chemicals. The Main model uses the primary confounder adjustment as given in the Methods. The “County Water Use Adjusted” model adds an adjustment for county-level water usage to the Main model. The “Min 2 Positives Tests” model uses the adjustment from the Main model but requires 2 positive (above Minimum Reporting Level) tests for a PFAS chemical before declaring that a positive test for that PFAS.

**Table 3.** Associations between PFAS and increases in chronic conditions via. cumulative link models

Exposure	Main model	Min 2 positive tests	County water use adjusted
Any PFAS	1.24 (1.11, 1.40)	1.24 (1.10, 1.39)	1.23 (1.09, 1.38)
PFOA	1.21 (1.07, 1.37)	1.22 (1.01, 1.47)	1.21 (1.07, 1.36)
PFHpA	1.27 (1.08, 1.50)	1.26 (1.04, 1.51)	1.24 (1.04, 1.47)

Results of the association between PFAS exposure and multimorbidity using cumulative link models are presented as the odds ratio with the 95% confidence interval in parentheses. In the “Exposure” column Any PFAS represents exposure to any of the four PFAS found in NC although PFOA and PFHpA refer to exposure to those specific PFAS chemicals. The Main model uses the primary confounder adjustment as given in the Methods. The “County Water Use Adjusted” model adds an adjustment for county-level water usage to the Main model. The “Min 2 Positives Tests” model uses the adjustment from the Main model but requires 2 positive (above Minimum Reporting Level) tests for a PFAS chemical before declaring that a positive test for that PFAS.

LCI indicates lower 95% confidence interval; OR, odds ratio; UCI = upper 95% confidence interval.

We also observed associations between multimorbidity and the two most prevalent PFAS (PFOA and PFHpA) with associations stronger for PFOA (OR = 1.30, 95% CI = 1.12, 1.52) than PFHpA (OR = 1.20, 95% CI = 0.97, 1.47). As with associations with any PFAS, these associations were also observed in the cumulative link model (**Table 3**).

To observe if health effects were potentially compounded by exposure to multiple PFAS, we examined if associations with multimorbidity increased for just PFOA or PFHpA as compared with potential exposure to both PFAS. In our study sample, 3,142 participants (30.9%) resided in a zip code exposed to one of PFOA or PFHpA, while 960 (9.44%) resided in zip codes exposed to both. We observed an OR of 1.24 (95% CI = 1.05, 1.45) for exposure to just one of the two PFAS but an OR of 1.38 (95% CI = 1.09, 1.76) for exposure to both PFAS, although he width of the confidence intervals precludes any strong statistical statement on any difference in the associations. The difference in association remained when adjusting for county water usage (eTable 5; http://links.lww.com/EE/A192).

**Figure 2.**
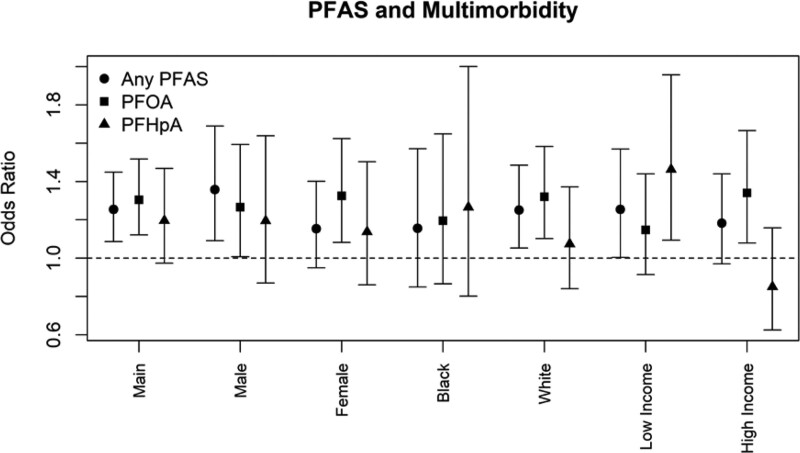
Stratified associations between PFAS exposure and Multimorbidity Stratified analyses showing the association between exposure to any PFAS (circles), PFOA (squares), and PFHpA (triangles) and multimorbidity via. logistic regression models. Confounder adjustment is given in the Methods. For stratified models the variable upon which the stratification is based was removed as a confounder in the model. Low Income refers to individuals residing in 2010 census block groups where the median household income was below the median of the distribution observed in the study cohort. High Income refers to individuals residing in 2010 census block groups where the median household income was above the median of the distribution observed in the study cohort. Error bars represent the 95% confidence interval.

Associations between multimorbidity and both PFOA and PFHpA were similar among both males and females. When stratifying on race, associations with multimorbidity were stronger among White study participants than Black participants. When stratifying on income, associations with multimorbidity were similar for any PFAS and for PFOA specifically, but multimorbidity associations with PFHpA were much stronger among study participants residing in lower income areas as compared with those residing in higher income areas (**Figure 2**). Finally, given the high prevalence of missing smoking information, we pooled associations from 5 imputations of smoking history and examined whether associations differed when smoking was imputed. Results with imputed smoking history were similar to results from the main model (eTable 6; http://links.lww.com/EE/A192).

### Individual chronic diseases and PFAS

As stated in the methods, we also examined associations with individual chronic diseases that had at least 300 cases in our study cohort. There were 9 chronic diseases fitting this definition (eTable 3; http://links.lww.com/EE/A192). We observed associations between exposure to any PFAS and hypertension, ischemic heart disease, arrhythmias, osteoporosis, and dyslipidemia (**Table 4**). When examining PFOA and PFHpA individually associations were largely similar as observed with any PFAS, with associations with hypertension being observed for both PFOA and PFHpA exposure (eTable 7; http://links.lww.com/EE/A192).

**Table 4.** Associations between exposure to any PFAS and individual chronic diseases

Outcome	OR (95% CI)	*P*
Arrhythmia	1.28 (1.04, 1.59)	0.02
Chronic kidney disease	1.14 (0.87, 1.51)	0.34
Dyslipidemia	1.19 (1.02, 1.39)	0.03
Heart failure	1.00 (0.74, 1.34)	0.99
Hypertension	1.32 (1.15, 1.52)	6.2 × 10^–5^
Ischemic heart disease	1.32 (1.05, 1.66)	0.02
Liver disease	0.93 (0.71, 1.21)	0.6
Osteoporosis	1.45 (1.05, 2.01)	0.03
Stroke	1.11 (0.78, 1.58)	0.57
Type 2 diabetes	1.05 (0.87, 1.26)	0.62

Only chronic diseases with at least 300 cases were examined. The exposure evaluated for all models was exposure to any of the four PFAS examined within this manuscript (PFOA, PFHpA, PFOS, PFHxS). Confounder adjustment for associations is given in the Methods.

CI inidcates confidence interval; OR, odds ratio.

## Discussion

PFAS exposure remains a national concern, and despite decades of research we are still working to uncover both the basic mechanisms of its toxicology as well as the public health effects of long-term exposure to this chemical class. In this study, we examined associations with exposure to PFAS compounds using electronic health records. Unlike many previous studies which focused on individual disease outcomes, we used the depth of clinical phenotyping available via. electronic health records to examine the co-occurrence of multiple chronic diseases, that is, multimorbidity, which has not yet been examined in relation to PFAS exposure. We observed strong associations with multimorbidity that were in part driven by individual disease associations observed in other epidemiologic studies. Thus, this study highlights not only known associations between PFAS and chronic disease, but the novel aspect that PFAS exposure might drive multimorbidity—compounding its health effects.

Lipid dysregulation has been perhaps the most consistent health effect associated with PFAS exposure in epidemiologic cohort studies. In a study of highly exposed residents and workers of a rural, primarily White (97%) community of the Mid-Ohio Valley, researchers reported an OR of 1.24 for hypercholesterolemia (95% CI: 1.15, 1.33) when comparing the lowest two quartiles of PFOA exposure to the upper quartile.^33^ Associations between PFOA exposure and elevated serum cholesterol have also been reported for nationally representative populations.^34^

Our study also found a strong association between PFOA exposure dyslipidemia with a confidence interval that overlapped that seen in previous studies (**Table 4**), further highlighting the impact of PFAS exposure on lipid metabolism and the consistence of this study with the published literature. Although overall consistent, differences in the point estimate between this study and previous could be attributed to the often more highly exposed populations in previous studies, demographic differences, study design differences, as well as simply random error in the point estimates.

Associations between PFAS and both hypertension and cardiovascular disease also reflected previously reported associations. In the case of cardiovascular disease, initial epidemiological evidence only showed associations in cross-sectional studies, however later longitudinal studies of carotid artery intima thickness also showed associations. There is also increasing experimental evidence linking PFAS exposure to platelet activation and thrombus formation.^11^ Our observed associations with arrhythmias have not been previously examined and may suggest a novel cardiac parameter worthy of further investigation. Outside of these individual chronic diseases, we also observed associations with osteoporosis. This association has also been observed in a nationally representative population.^35^ As one of the first studies to use EHRs to study PFAS exposure, the consistency of these associations with previous epidemiologic studies, particularly nationally representative studies, highlights the ability of EHRs to power future research efforts into this important chemical class.

The primary focus of this study was to examine the effect of PFAS chemicals, primarily PFOA and PFHpA, on multimorbidity. Although numerous studies have identified associations between various PFAS and individual health outcomes, this is to our knowledge the first study to specifically quantify its effects on multimorbidity. Associations with multimorbidity may better allow researchers to quantify the total health effects of PFAS chemicals. These associations may also guide research into the mechanisms of action of PFAS by better indicating the constellation of health effects, some of which may share a common PFAS-relevant pathophysiologic mechanism. One of the primary mechanism of actions proposed for PFAS is through the nuclear receptor pathway, particularly PPARα.^15,16^ In addition, FAS exposure is associated with increased inflammatory markers and oxidative stress.^14,36^ Given the systemic effects which can be induced by both oxidative stress and PPARα dysregulation, the combination of these two molecular actors may in part explain the broad health effects observed in association with PFAS exposure.

### Strengths and limitations

There are several strengths and limitations of this study. One of the primary strengths is the use of EHRs, which allowed us to collect a large population of individuals with comprehensive assessment of chronic disease. There has been one previous study of PFAS exposure using electronic health records. This previous study examined women only and focused on exposures occurring within a single small town and did no confounder adjustment or stratifications. Thus, our study provides significant improvements though both studies were able to replicate several previously observed associations.^27^ In this study, we examined a random sample of individuals; however, they are still all hospital patients and thus are more likely to be older and have pre-existing health conditions than the general population. Despite this, associations observed here match associations seen in other large, population-based epidemiologic studies highlighting the generalizability of PFAS health effects and the potential utility of EHRs for PFAS studies. A limitation of our study is that it was a cross-sectional analysis, meaning that causality is very difficult to infer; however, this study design was necessary due to the co-occurrence of outcome determination and PFAS testing. Future studies that are able to reconstruct historical PFAS exposures in the context of an EHR cohort may be able to repeat these analyses using incident disease definitions.

Although we were able to adjust for a broad range of confounders using both the medical record as well as public data, there is always the possibility of unmeasured confounding. This is particularly true for medical records which often do not capture individual-level socioeconomic status. Although our study recapitulates results observed in epidemiologic studies which did adjust for individual-level SES, replicating the multimorbidity associations in a similar cohort with even broader confounder adjustment would be an important validation step. The use of electronic health records is also what underlined our ability to estimate multimorbidity using 16 different chronic diseases. That depth of clinical disease history phenotyping is often unavailable in more targeted cohort studies. However, we do acknowledge that our disease outcomes were defined based on ICD-9/10 codes, which can be a limited means of disease assessment. However, ICD codes still remain a common means of assessing disease outcomes and represent the most comprehensive assessment using the data available. In addition, the potential for access to the hospital to impact observed diagnoses is a very real possibility. Those with greater access (higher incomes, insurance status) may be more likely to have conditions diagnosed early. We adjusted for socioeconomic status, urbanicity, and county-level indicators of access to healthcare in all models. We also stratified on income as a means of examining the potential impact of socioeconomic status on our associations.

This study primarily focused on PFAS exposure through drinking water, which is not the only route of PFAS exposure. Airborne, dust, and food exposures may all contribute to PFAS exposure. However, there are no geospatially precise, systematic maps of these exposures, which would allow them to be utilized for population studies such as this one. This study still provides key health information associated with primary route of exposure that is of strong concern to many communities.

Drinking water PFAS contamination is unfortunately not uniformly assessed in the United States. We used the UCMR3, a national monitoring program performed by the US Environmental Protection Agency for drinking water contamination in large public water systems. This gave us the breadth of PFAS monitoring necessary for this study, however, NC had a limited exposure contrast and we did not have access to uniform well water testing throughout the state. There were also some zip codes which were not served by a water system assessed in the UCMR3 and thus were not able to be included in the study as no information on the level of contamination or lack thereof. As the UCMR3 attempted to do a systematic nationwide assessment of water systems, we do not expect systematic biases between those water systems assessed and those not assessed except for the potential increased use of well water among the zip codes not included. Another potential limitation is that zip code-level estimates of aggregate exposures will not reflect the variation in individual exposures to these chemicals; however, systemic individual-level chemical testing is not available in resources like electronic health records. Another limitation is that we did not have PFAS testing data from before 2013, as is the case with many communities throughout the United States. A 2013 study^37^ found highly elevated level of PFAS contamination in North Carolina water samples taken downstream from a long operating fluorochemical manufacturer suggesting a long history of the presence of PFAS in North Carolina waterways, and thus drinking water. This would match nationwide data suggesting a long history of ubiquitous PFAS exposures in the United States,^2^ which we would not expect to be different for North Carolina. Despite these limitations, we were able to observe consistent associations, that stood up to adjustment for county-level drinking water usage as well as increased stringency of our PFAS determinations by requiring multiple positive tests. Although not without their limitations, regional, and nationwide maps of PFAS testing—perhaps in combination with improved exposure reconstruction methods—can allow a host of existing human cohort resources to be used to study this pressing environmental health need.

In conclusion, we observed the exposure to PFAS chemicals, in particular PFOA and PFHpA, is associated with multimorbidity. This study also shows that electronic health record cohorts can recapitulate associations with PFAS chemicals observed in population-based epidemiologic cohort studies, establishing them as a new route to study this important chemical class. Future studies should expand on the impact of PFAS exposure on multiple chronic diseases to fully capture the public health implications of this chemical exposure.

## Conflict of interest

C.W.-C. is a scientific advisor for the Clock Foundation. The Clock Foundation had no role in any aspect of this work. The remaining authors declare that they have no conflicts of interest with regard to the content of this report.

## Source of funding

This work was funded by the US Environmental Protection Agency. The research described in this article has been reviewed by the Center for Public Health and Environmental Assessment, US Environmental Protection Agency, and approved for publication. Approval does not signify that the contents necessarily reflect the views and policies of the Agency, nor does mention of trade names or commercial products constitute endorsement or recommendation for use. The project described was supported by the National Center for Advancing Translational Sciences (NCATS), National Institutes of Health, through Grant Award Number UL1TR002489. The content is solely the responsibility of the authors and does not necessarily represent the official views of the NIH.

## Supplementary Material


