# Supplementary material for: Associations between PFAS occurrence and multimorbidity as observed in an electronic health record cohort
Source: Environ Epidemiol. 2022 Jul 14;6(4):e217. doi: 10.1097/EE9.0000000000000217 (PMC9374186; doi:10.1097/EE9.0000000000000217)
Supplement: Supplementary file 1 [file ee9-6-e217-s001.docx]

**Supplemental Digital Content Figures and Tables**


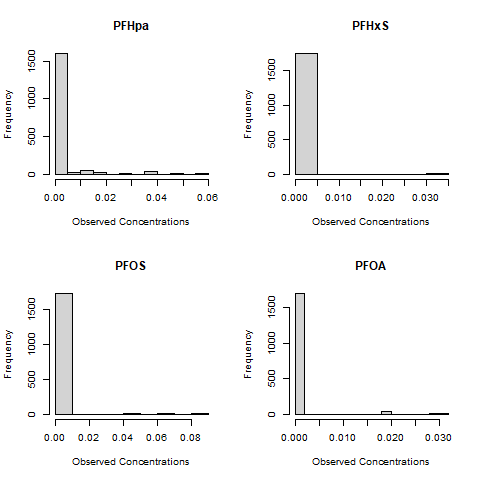


eFigure 1. Distribution of Observed PFAS chemical concentrations

Show is the distribution of PFAS concentrations for the four PFAS testing positive at least once for a water system serving a zip code in our study sample. For plotting purposes observations below the minimum reporting level for each PFAS were set to 0. As can be seen each PFAS was seen in a minority of zip codes with PFHpA being seen the least often. And this distribution of observed test values was certainly not smooth or continuous. This motivated our decision to use a binary indicator of a water system testing positive (above the minimum reporting level) for analyses.

eFigure 2. Distribution of PFOA and PFHpA in NC by zip code

Shown are the zip codes where a public water system serving that zip code tested positive for PFOA (top) or PFHpA (bottom) during the Unregulated Contaminant Monitoring Rule 3 testing. In grey are the zip codes that were not served by any public water system tested during the Unregulated Contaminant Monitoring Rule 3 testing.

| Outcome | ICD-9 | ICD-10 |
| --- | --- | --- |
| Type 2 Diabetes | 250.* | E11.* |
| Hypertension | 401.* | I10.* |
| Chronic Kidney Disease | 585.* | N18.* |
| Dyslipidemia | 272.* | E78.* |
| Peripheral Arterial Disease | 443.* | I73.* |
| Chronic Obstructive Pulmonary Disease | 491.*, 492.* | J43.*, J44.* |
| Ischemic Heart Disease | 414.* | I2[0-5].* |
| Heart Failure | 428.* | I50.* |
| Arrhythmia | 427.* | I49.* |
| Stroke | 430.*, 431.*, 433.[0-3], 434.*, 435.*, 436.* | I63.* |
| Breast Cancer | 174.*, 175.0 | C79.81, C20.* |
| Colorectal Cancer | 153.*, 154.* | C18.*, C20.* |
| Prostate Cancer | 185.*, 233.4, V10.46 | C61.*, Z85.46 |
| Lung Cancer | 162.* | C43, V10.91, Z85.118 |
| Osteoporosis | 733.* | M8[0-1].* |
| Liver Disease | 570.*, 571.[4-9], 572.*, 573.* | K7[0-4].* |

eTable 1. Definitions of Chronic Diseases that Defined Multimorbidity.

Multimorbidity was defined as the presence of 2 or more of the above chronic diseases based on the listed ICD-9 and ICD-10 definitions.

| Variable | Mean in Study sample | SD in study sample |
| --- | --- | --- |
| Average annual health care cost ($) | 8859.47 | 920.23 |
| Food Environment Index | 6.87 | 0.77 |
| Mortality rate for drug poisoning deaths | 10.17 | 3.33 |
| Number of Dentists per 100,000 people | 60.92 | 48.90 |
| Number of mental health providers per 100,000 people | 201.92 | 160.84 |
| Number of non-primary care physicians per 100,000 people | 81.12 | 47.32 |
| Number of Primary Care Physicians per 100,000 people | 79.28 | 44.78 |
| Number of social associations (organizations) per 10,000 people | 11.07 | 1.82 |
| Number of violent crimes per 100,000 people | 297.77 | 150.78 |
| Percent Adult Smokers | 18.56 | 5.51 |
| Percent Adults Physically Inactive | 23.36 | 5.17 |
| Percent Adults with Obesity (body mass index > 30) | 28.58 | 4.73 |
| Percent of Single Parent Households | 34.00 | 7.57 |
| Percent Population Unemployed | 18.47 | 2.76 |
| Percent Residents with Access to Exercise Opportunities | 76.48 | 15.46 |
| Percentage of households with at least 1 of 4 housing problems: overcrowding, high housing costs, or lack of | 16.25 | 2.22 |
| Percentage of population unemployed | 7.30 | 1.72 |
| Percentage of population with limited access to healthy foods | 6.10 | 3.56 |
| Ratio of Income in the upper quintile to income in the lower quintile | 4.76 | 0.66 |

eTable 2. Distribution of County-level Variables

Distribution of the 19 county-level variables from the County Health Rankings used as confounders in the models

| Chronic Disease | N | % |
| --- | --- | --- |
| Hypertension | 2556 | 25.1 |
| Dyslipidemia | 1762 | 17.3 |
| Type 2 Diabetes | 1068 | 10.5 |
| Arrhythmia | 852 | 8.38 |
| Chronic Kidney Disease | 460 | 4.52 |
| Liver Disease* | 456 | 4.48 |
| Heart Failure | 436 | 4.29 |
| Osteoporosis | 330 | 3.25 |
| Stroke | 305 | 3.00 |
| Peripheral Arterial Disease | 278 | 2.73 |
| Chronic Obstructive Pulmonary Disease | 180 | 1.77 |
| Female Breast Cancer | 160 | 1.57 |
| Prostate Cancer | 146 | 1.44 |
| Lung Cancer | 107 | 1.05 |
| Colorectal Cancer | 69 | 0.68 |
| Ischemic Heart Disease | 742 | 7.30 |

eTable 3. Frequency of chronic diseases observed in the study population

| N = 15,118 | Mean | SD |
| --- | --- | --- |
| Age (y) | 51.7 | 18.1 |
| Percent Urbanicity | 70.8 | 38.5 |
| Median Home Value ($) | 210609 | 118618 |
| Percent Households Below Federal Poverty Level | 16.8 | 14.7 |
| Percent of Homes Receiving Public Assistance | 1.82 | 2.80 |
| Observation Time (y) | 7.84 | 4.28 |
|  | N | % |
| Females | 10815 | 62.4 |
| Males | 6507 | 37.6 |
| Race - White | 10976 | 63.4 |
| Race - Black | 4485 | 25.9 |
| Race - Other | 1861 | 10.7 |
| Never Smoker | 5259 | 30.4 |
| Former Smoker | 2584 | 14.9 |
| Current Smoker | 2045 | 11.8 |
| Unknown Smoking Status | 7434 | 42.9 |
| Multimorbidity | 4113 | 23.7 |
| PFOA present | 5235 | 30.2 |
| PFHpA present | 3361 | 19.4 |
| Any PFAS present | 7237 | 41.8 |

eTable 4. Characteristics of original population before restriction to individuals reporting only one address. Percent Urbanicity, Median Home Value, Percent Households Below Federal Poverty Level, and Percent Homes Receiving Public Assistance all assessed using the 2010 US Census at the block group level. SD = standard deviation

| Model | Number PFAS exposed to | OR | LCI | UCI |
| --- | --- | --- | --- | --- |
| Logistic | 1 | 1.24 | 1.05 | 1.45 |
| Logistic | 2 | 1.38 | 1.09 | 1.76 |
| Logistic - Water Usage Adjusted | 1 | 1.20 | 1.01 | 1.41 |
| Logistic - Water Usage Adjusted | 2 | 1.37 | 1.08 | 1.74 |
|  |  |  |  |  |
| CLM | 1 | 1.20 | 1.06 | 1.37 |
| CLM | 2 | 1.34 | 1.10 | 1.63 |
| CLM - Water Usage Adjusted | 1 | 1.18 | 1.03 | 1.35 |
| CLM - Water Usage Adjusted | 2 | 1.33 | 1.09 | 1.62 |

eTable 5. Associations between multimorbidity and increasing number of PFAS

The “Number of PFAS exposed to” column refers to the number of distinct PFAS for which all the public water systems included in the UCMR3 testing tested positive for. The primary adjustment for all models adjusted for age at HF diagnosis, sex, race, smoking status (current/former/never), percent urbanicity at the census block group, median household value at the census block group, percent homes under the federal poverty line at the census block group, and percent of homes receiving public assistance at the census block group, and 19 county-level indicators of socioeconomic status and access to health care (**eTable 2**) as described in the Methods. In the “Model” column “Logistic” refers to the logistic regression models on the binary multimorbidity definition; “Logistic – Water Usage Adjusted” refers to adjusting for county water level usage in the logistic regression models in addition to the aforementioned confounders; “CLM” refers to the cumulative link models which model the increase in the number of chronic conditions as a function of PFAS exposure using the same adjustment as the logistic regression models; and “CLM – Water Usage Adjusted” refers to the addition of county-level water usage to the aforementioned adjustment for the cumulative link models. LCI = lower 95% confidence interval; OR = odds ratio; LCI = upper 95% confidence interval

| Model | Exposure | OR | LCI | UCI |
| --- | --- | --- | --- | --- |
| Logistic | Any PFAS | 1.25 | 1.08 | 1.44 |
| Logistic | PFOA | 1.31 | 1.12 | 1.52 |
| Logistic | PFHpA | 1.19 | 0.97 | 1.46 |
| Logistic | PFOA or PFHpA | 1.27 | 1.10 | 1.47 |
|  |  |  |  |  |
| CLM | Any PFAS | 1.23 | 1.10 | 1.38 |
| CLM | PFOA | 1.21 | 1.07 | 1.36 |
| CLM | PFHpA | 1.26 | 1.07 | 1.49 |
| CLM | PFOA or PFHpA | 1.23 | 1.09 | 1.38 |

eTable 6. Associations between PFOA, PFHpA and Multimorbidity with Imputed Smoking

For these models unknown smoking status was imputed as described in the methods. Results presented are the pooled estimates from 5 imputations of smoking status. All models were adjusted for age at HF diagnosis, sex, race, smoking status (current/former/never), percent urbanicity at the census block group, median household value at the census block group, percent homes under the federal poverty line at the census block group, and percent of homes receiving public assistance at the census block group, and 19 county-level indicators of socioeconomic status and access to health care (**eTable 2**) as described in the Methods. “Logistic” refers to the logistic regression models treating multimorbidity as a binary variable. “CLM” refers to the cumulative link models which model the increase in the number of chronic conditions using the same adjustment as the logistic regression model. LCI = lower 95% confidence interval; OR = odds ratio; UCI = upper 95% confidence interval

| Outcome | Exposure | OR | LCI | UCI | P |
| --- | --- | --- | --- | --- | --- |
| Arrhythmia | Any PFAS | 1.28 | 1.04 | 1.59 | 0.020223 |
| Arrhythmia | PFHpA | 1.27 | 0.92 | 1.76 | 0.152336 |
| Arrhythmia | PFOA | 1.30 | 1.05 | 1.62 | 0.01704 |
| Chronic Kidney Disease | Any PFAS | 1.14 | 0.87 | 1.51 | 0.341859 |
| Chronic Kidney Disease | PFHpA | 0.91 | 0.60 | 1.37 | 0.653505 |
| Chronic Kidney Disease | PFOA | 1.29 | 0.98 | 1.71 | 0.073326 |
| Dyslipidemia | Any PFAS | 1.19 | 1.02 | 1.39 | 0.029252 |
| Dyslipidemia | PFHpA | 1.17 | 0.93 | 1.48 | 0.173618 |
| Dyslipidemia | PFOA | 1.13 | 0.96 | 1.33 | 0.135803 |
| Heart Failure | Any PFAS | 1.00 | 0.74 | 1.34 | 0.986502 |
| Heart Failure | PFHpA | 1.02 | 0.64 | 1.63 | 0.928082 |
| Heart Failure | PFOA | 1.06 | 0.78 | 1.43 | 0.715804 |
| Hypertension | Any PFAS | 1.32 | 1.15 | 1.52 | 6.21E-05 |
| Hypertension | PFHpA | 1.35 | 1.11 | 1.65 | 0.002909 |
| Hypertension | PFOA | 1.25 | 1.08 | 1.45 | 0.002224 |
| Ischemic Heart Disease | Any PFAS | 1.32 | 1.05 | 1.66 | 0.019681 |
| Ischemic Heart Disease | PFHpA | 1.59 | 1.12 | 2.25 | 0.00968 |
| Ischemic Heart Disease | PFOA | 1.26 | 1.00 | 1.60 | 0.050893 |
| Liver Disease | Any PFAS | 0.93 | 0.71 | 1.21 | 0.598563 |
| Liver Disease | PFHpA | 0.90 | 0.60 | 1.34 | 0.600981 |
| Liver Disease | PFOA | 1.04 | 0.79 | 1.37 | 0.764652 |
| Osteoporosis | Any PFAS | 1.45 | 1.05 | 2.01 | 0.025803 |
| Osteoporosis | PFHpA | 1.20 | 0.73 | 1.98 | 0.477942 |
| Osteoporosis | PFOA | 1.46 | 1.04 | 2.04 | 0.028116 |
| Stroke | Any PFAS | 1.11 | 0.78 | 1.58 | 0.57318 |
| Stroke | PFHpA | 1.34 | 0.71 | 2.53 | 0.369715 |
| Stroke | PFOA | 1.11 | 0.78 | 1.60 | 0.553812 |
| Type 2 Diabetes | Any PFAS | 1.05 | 0.87 | 1.26 | 0.618226 |
| Type 2 Diabetes | PFHpA | 1.16 | 0.89 | 1.52 | 0.273618 |
| Type 2 Diabetes | PFOA | 1.13 | 0.93 | 1.37 | 0.223597 |

eTable 7. Associations between PFAS exposure and Chronic Disease

All models were adjusted for age at HF diagnosis, sex, race, smoking status (current/former/never), percent urbanicity at the census block group, median household value at the census block group, percent homes under the federal poverty line at the census block group, and percent of homes receiving public assistance at the census block group, and 19 county-level indicators of socioeconomic status and access to health care (**eTable 2**) as described in the Methods. Only chronic diseases with at least 300 cases were considered. LCI = lower 95% confidence interval; OR = odds ratio; UCI = upper 95% confidence interval
